# Supplementary figures and images for: 4G cloning: rapid gene assembly for expression of multisubunit protein complexes in diverse hosts
Source: Life Sci Alliance. 2024 Dec 2;8(1):e202402899. doi: 10.26508/lsa.202402899 (PMC11612967; doi:10.26508/lsa.202402899)

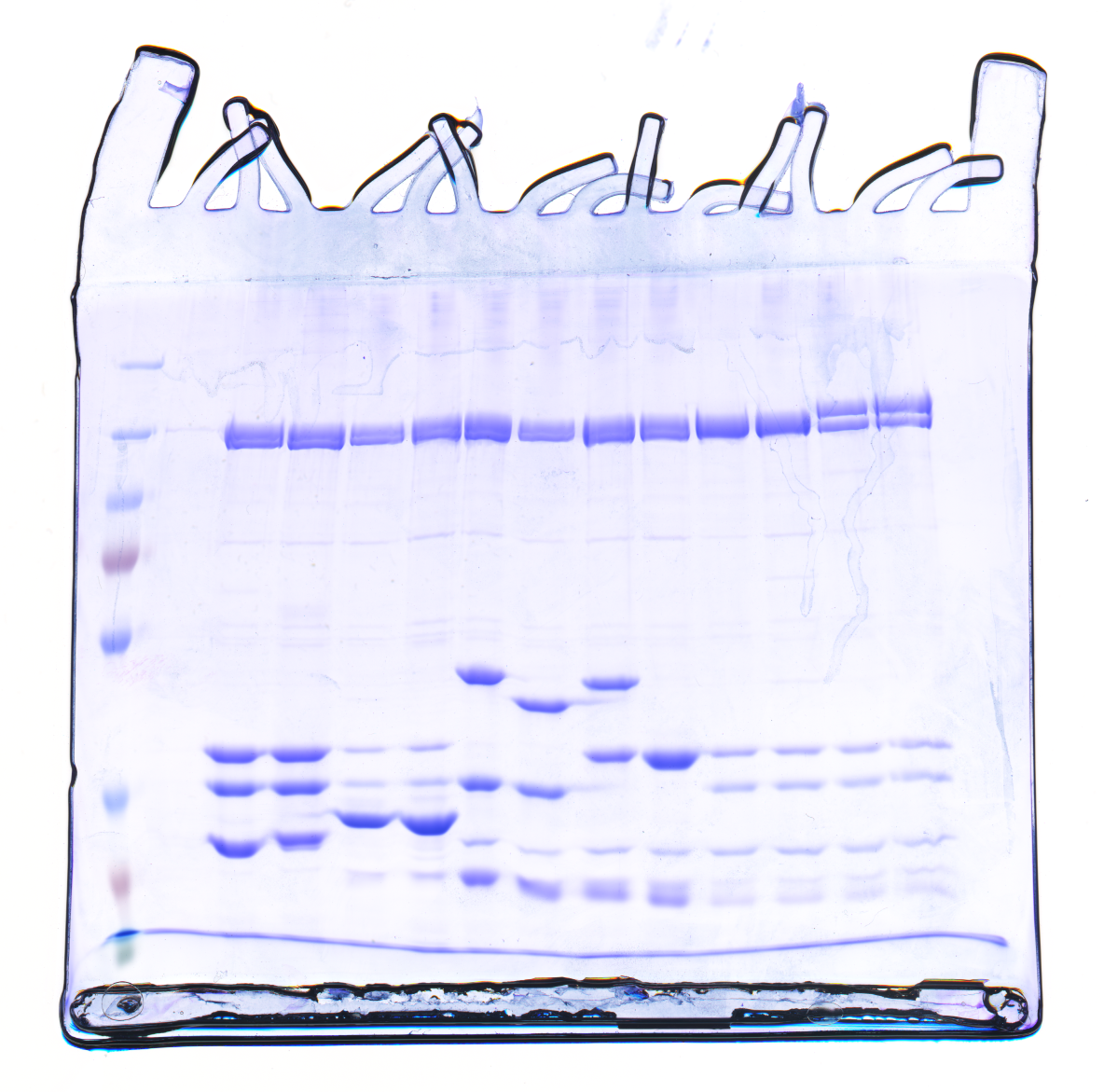

Supplement: Supplementary file 1 [file LSA-2024-02899_SdataF2_F3_F4_F5.zip › original data/Fig 4A _ Sphexamer screen.tif]

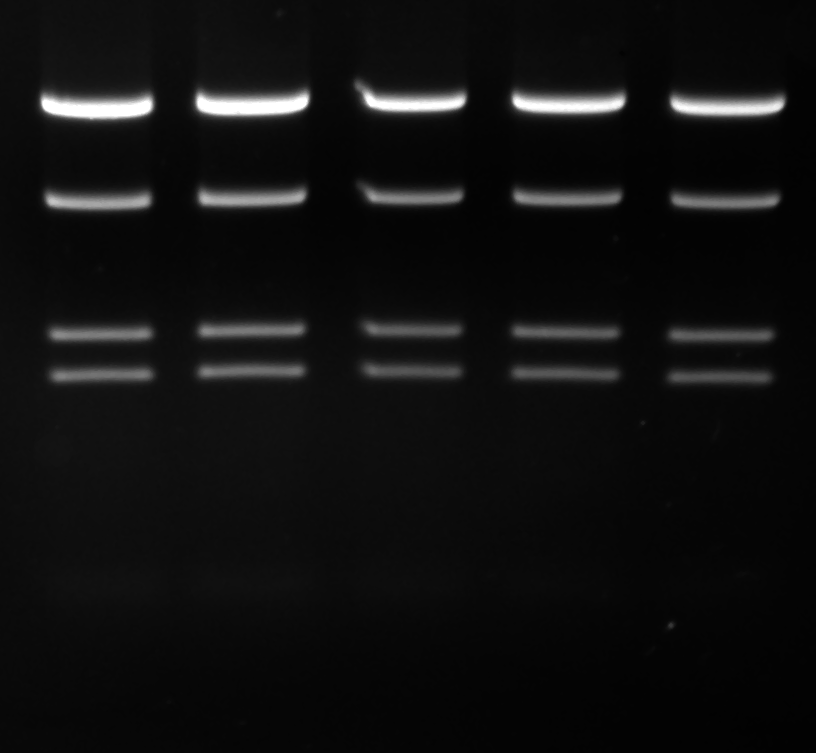

Supplement: Supplementary file 1 [file LSA-2024-02899_SdataF2_F3_F4_F5.zip › original data/Fig 2A _ 2 insert gel.png]

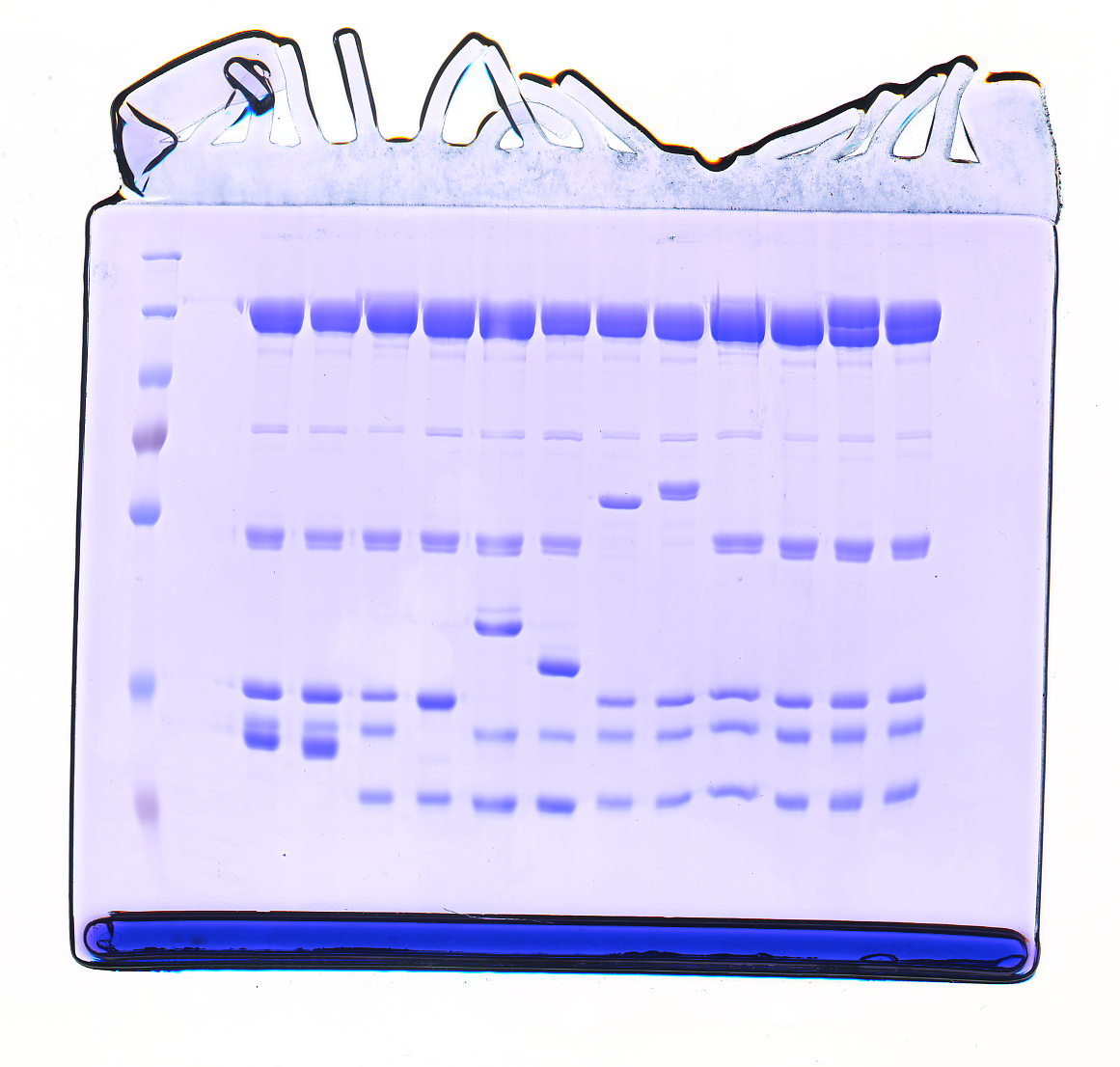

Supplement: Supplementary file 1 [file LSA-2024-02899_SdataF2_F3_F4_F5.zip › original data/Fig 4B _ Hshexamer screen.tif]

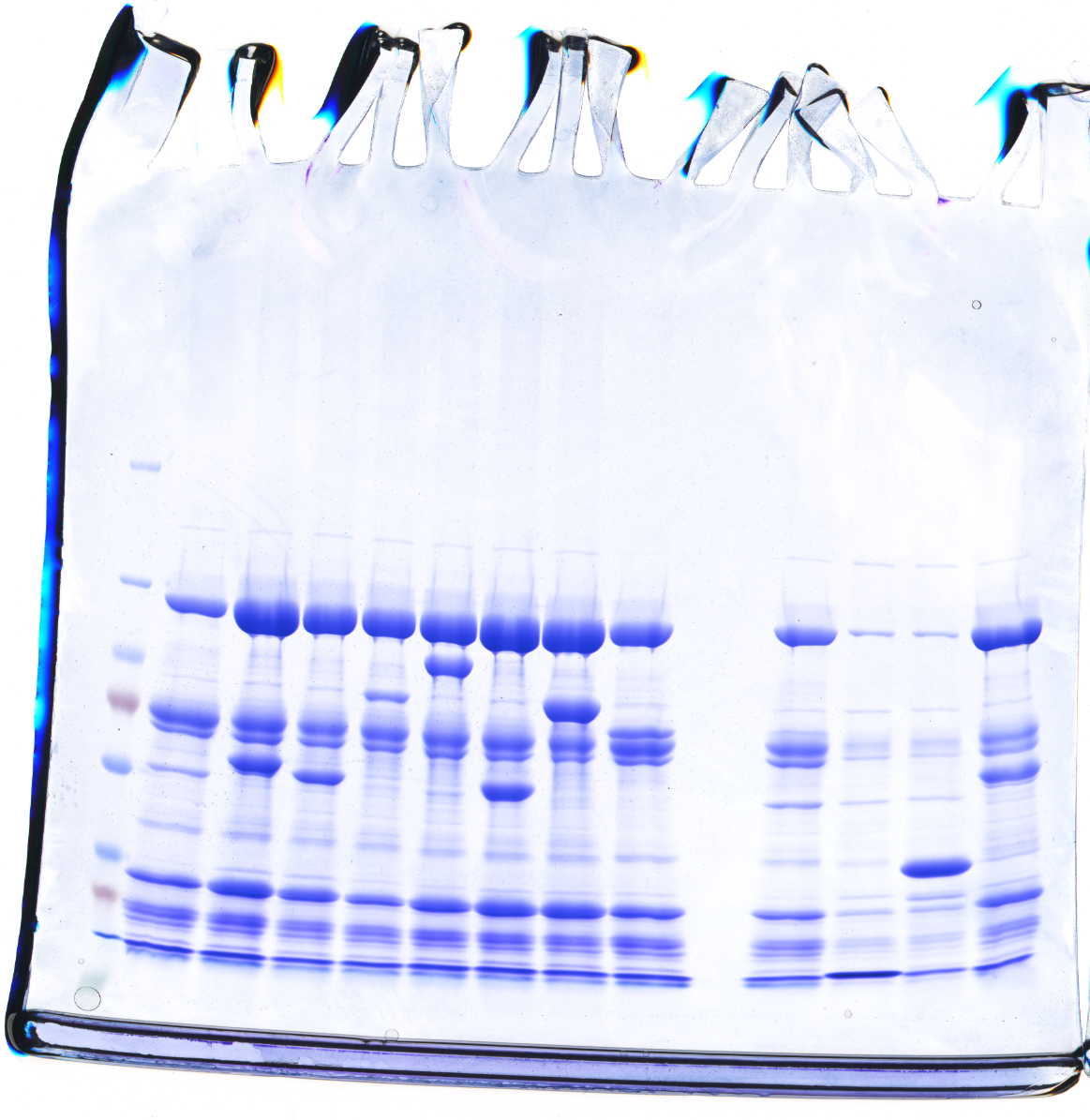

Supplement: Supplementary file 1 [file LSA-2024-02899_SdataF2_F3_F4_F5.zip › original data/Fig 3D _ Jet screen.tif]

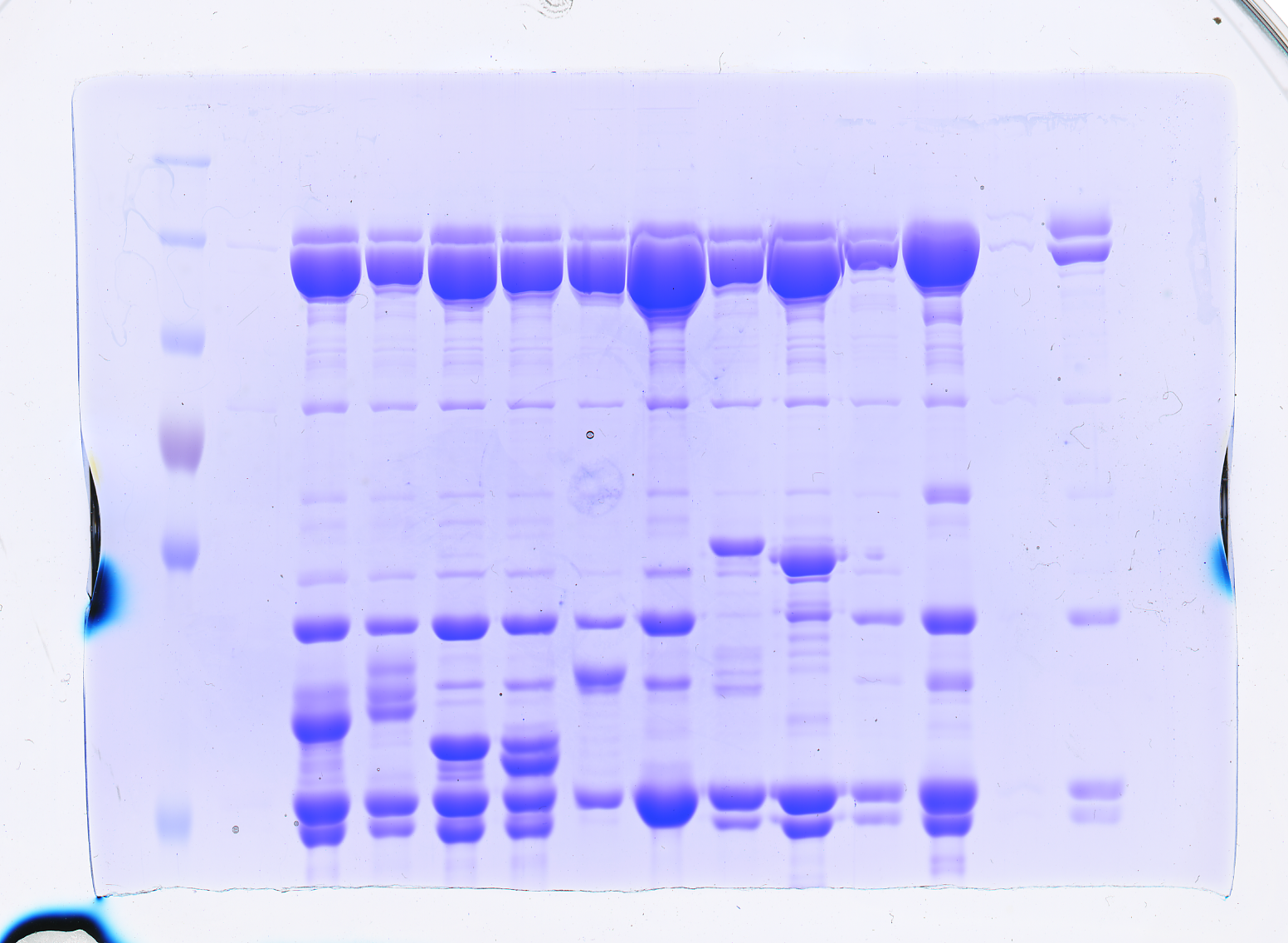

Supplement: Supplementary file 1 [file LSA-2024-02899_SdataF2_F3_F4_F5.zip › original data/Fig 3B _ Schexamer screen.tif]

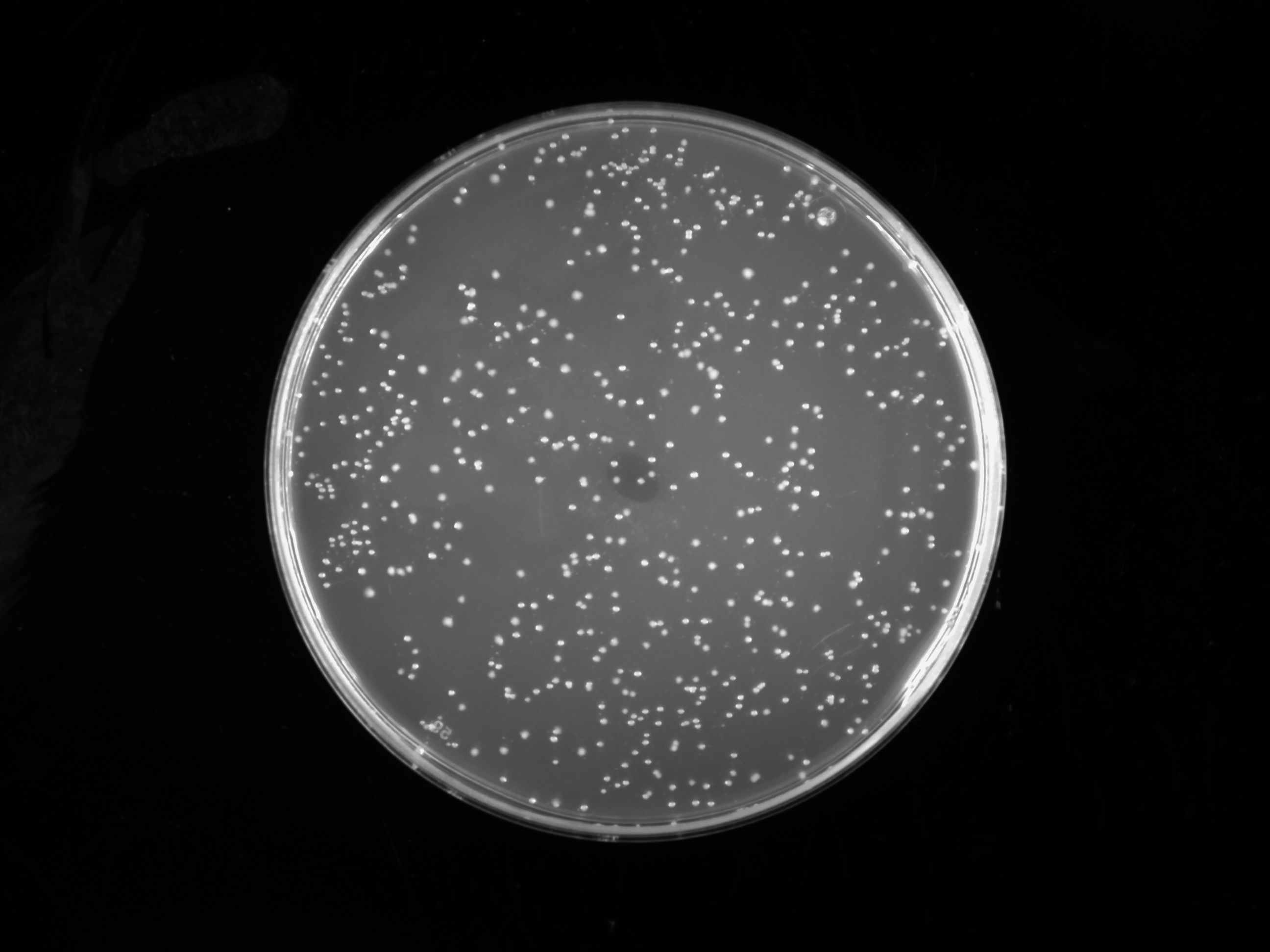

Supplement: Supplementary file 1 [file LSA-2024-02899_SdataF2_F3_F4_F5.zip › original data/Fig 2A _ 2 insert plate.tif]

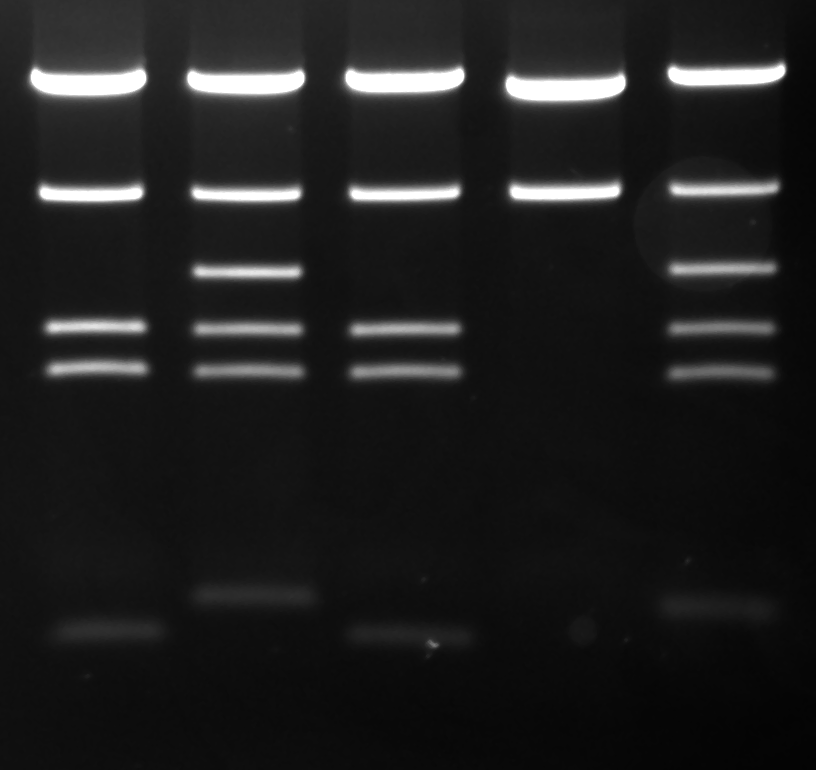

Supplement: Supplementary file 1 [file LSA-2024-02899_SdataF2_F3_F4_F5.zip › original data/Fig 2A _ 4 insert gel.png]

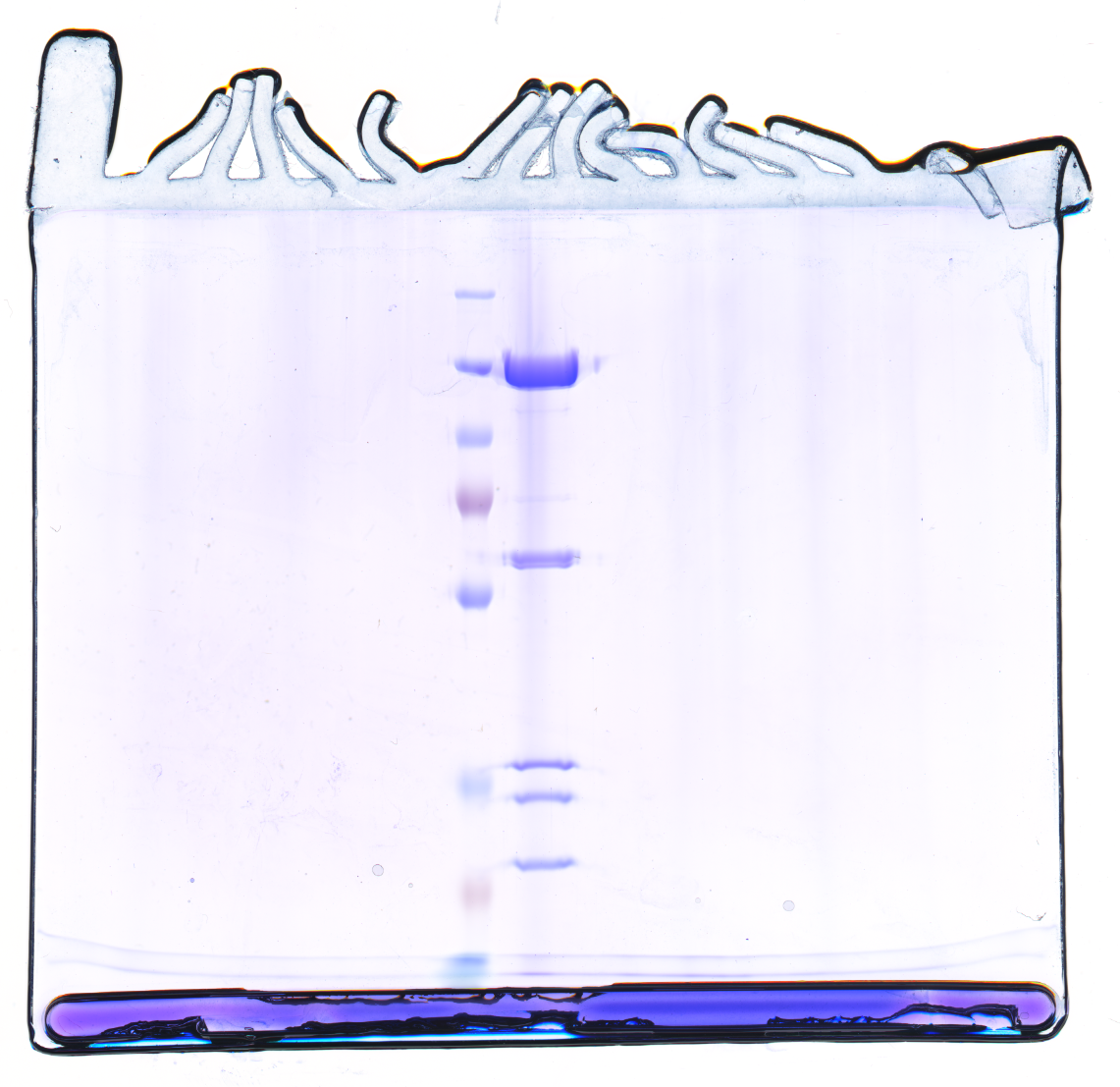

Supplement: Supplementary file 1 [file LSA-2024-02899_SdataF2_F3_F4_F5.zip › original data/Fig 5C _ Hshexamer purification single lane.tif]

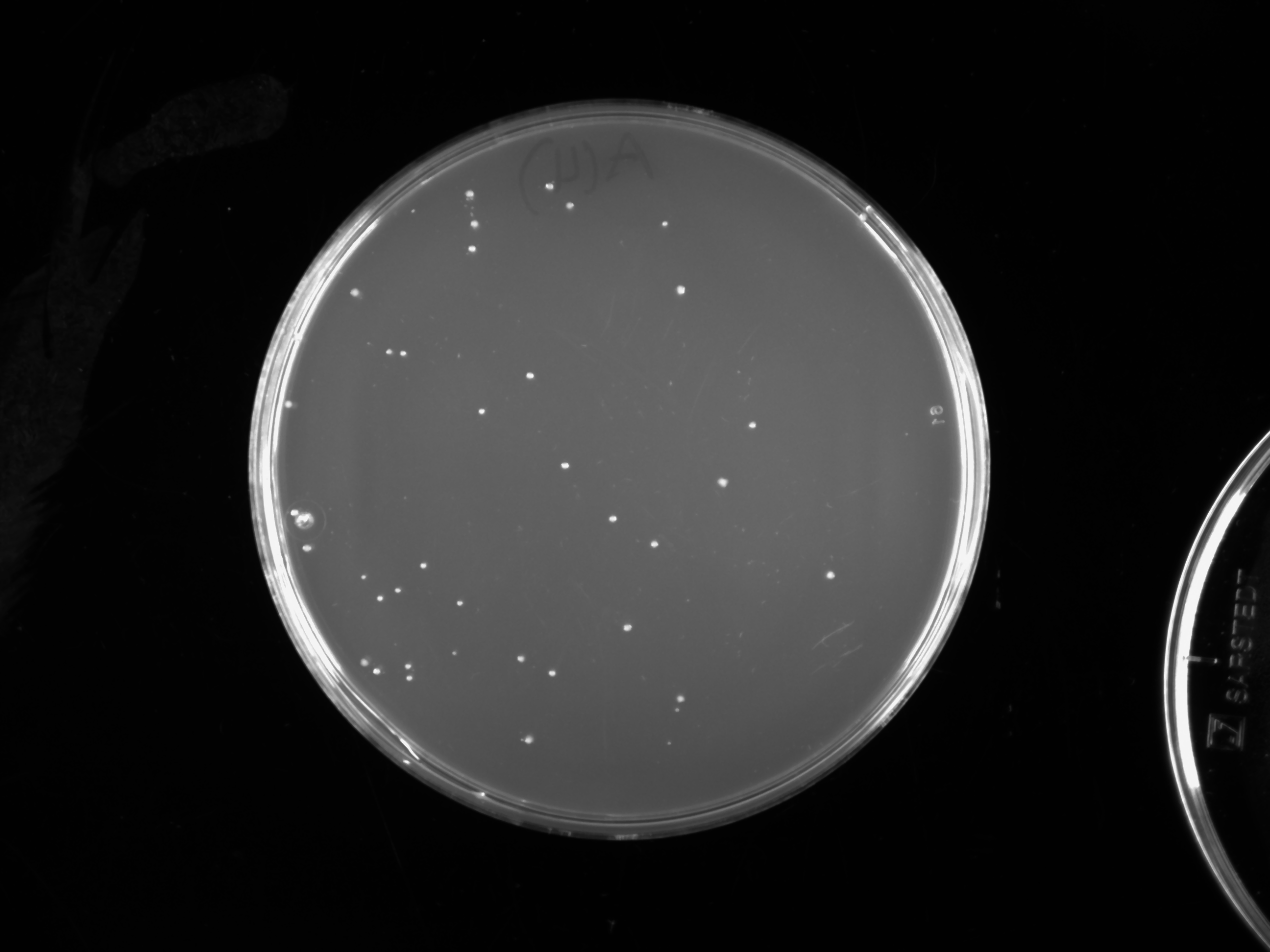

Supplement: Supplementary file 1 [file LSA-2024-02899_SdataF2_F3_F4_F5.zip › original data/Fig 2A _ 4 insert plate.tif]

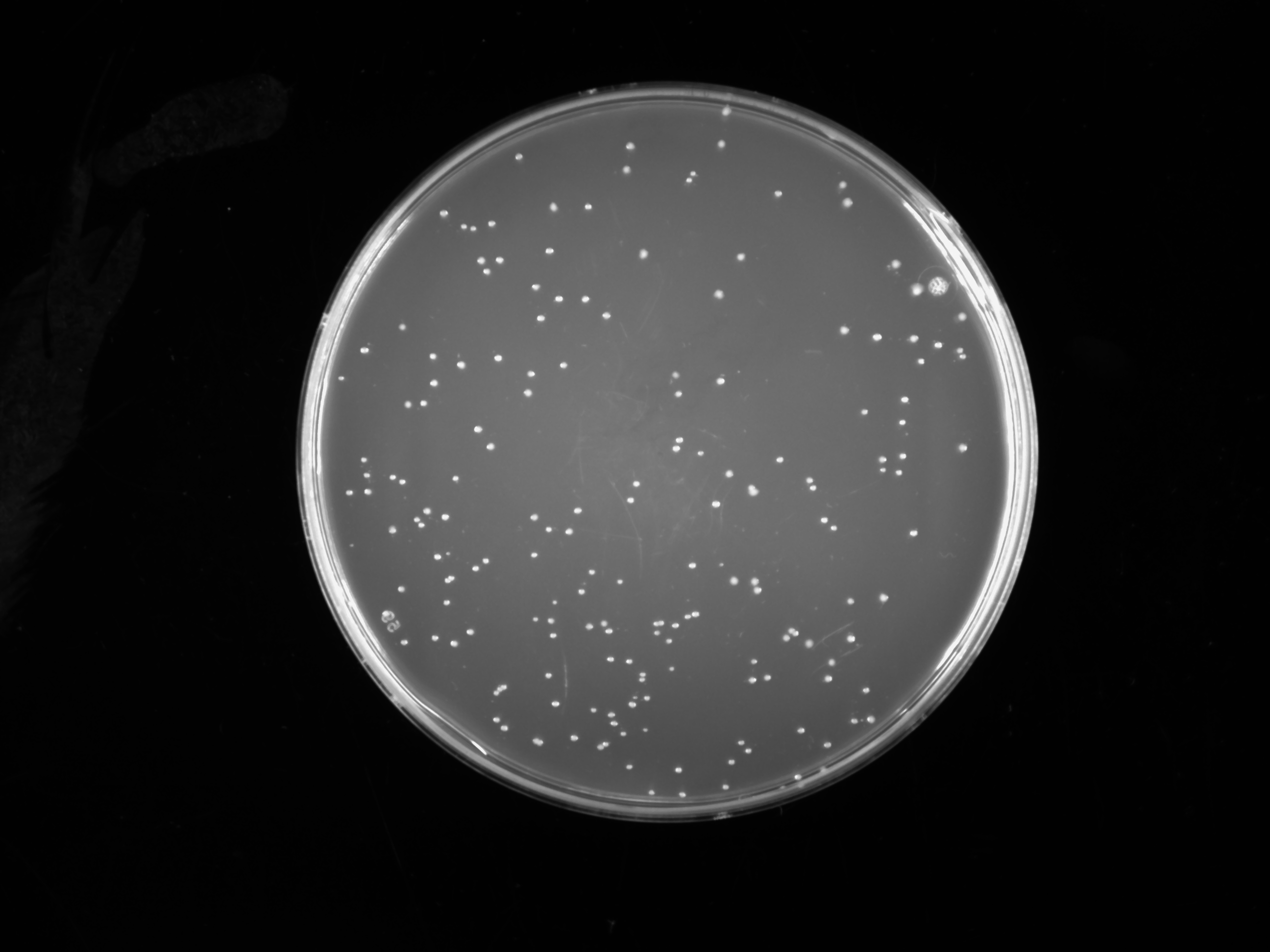

Supplement: Supplementary file 1 [file LSA-2024-02899_SdataF2_F3_F4_F5.zip › original data/Fig 2A _ 3 insert plate.tif]

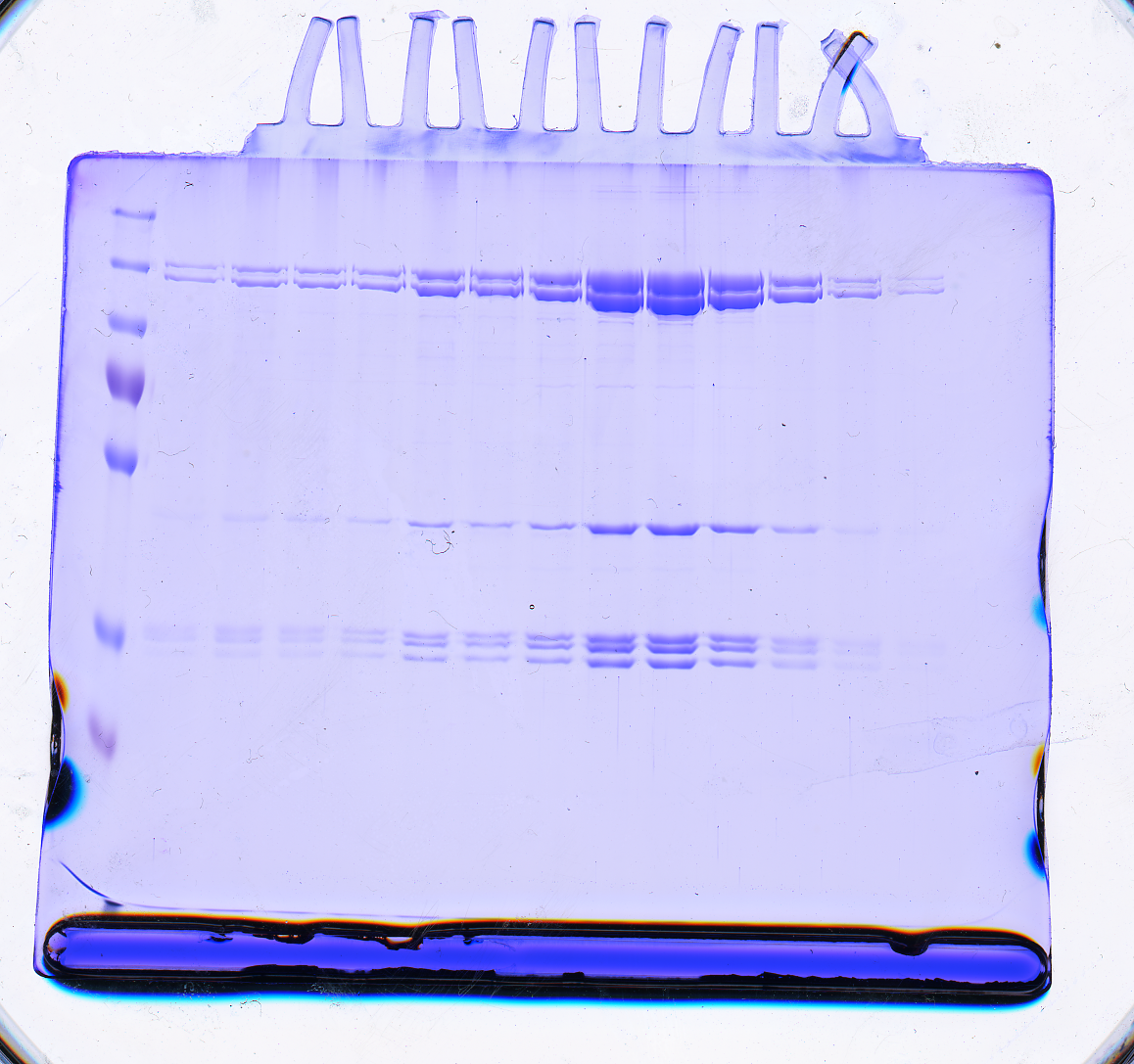

Supplement: Supplementary file 1 [file LSA-2024-02899_SdataF2_F3_F4_F5.zip › original data/Fig 5A _ Schexamer purification full gel.tif]

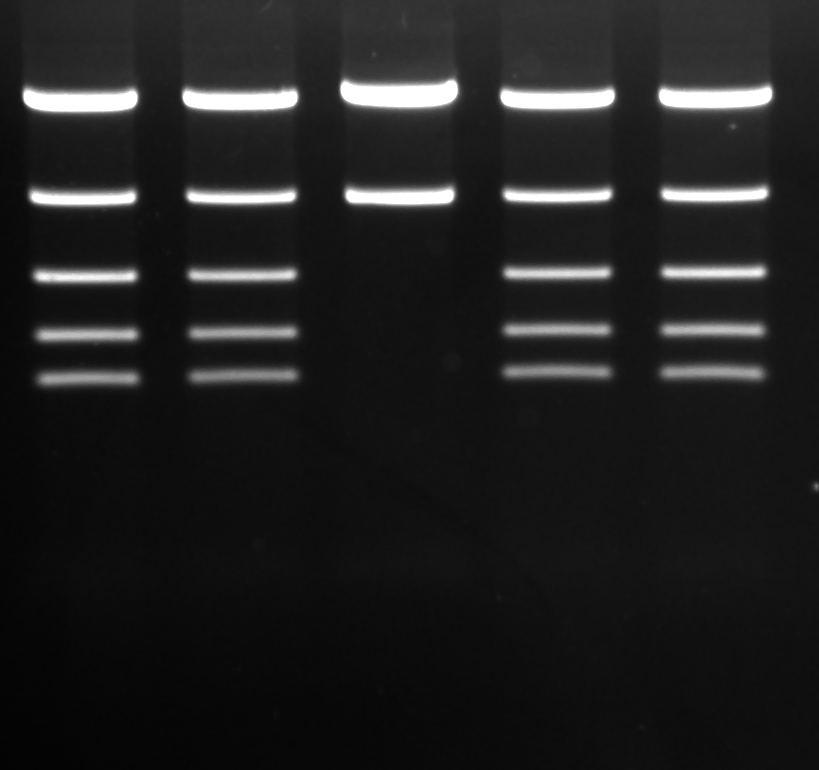

Supplement: Supplementary file 1 [file LSA-2024-02899_SdataF2_F3_F4_F5.zip › original data/Fig 2A _ 3 insert gel.png]

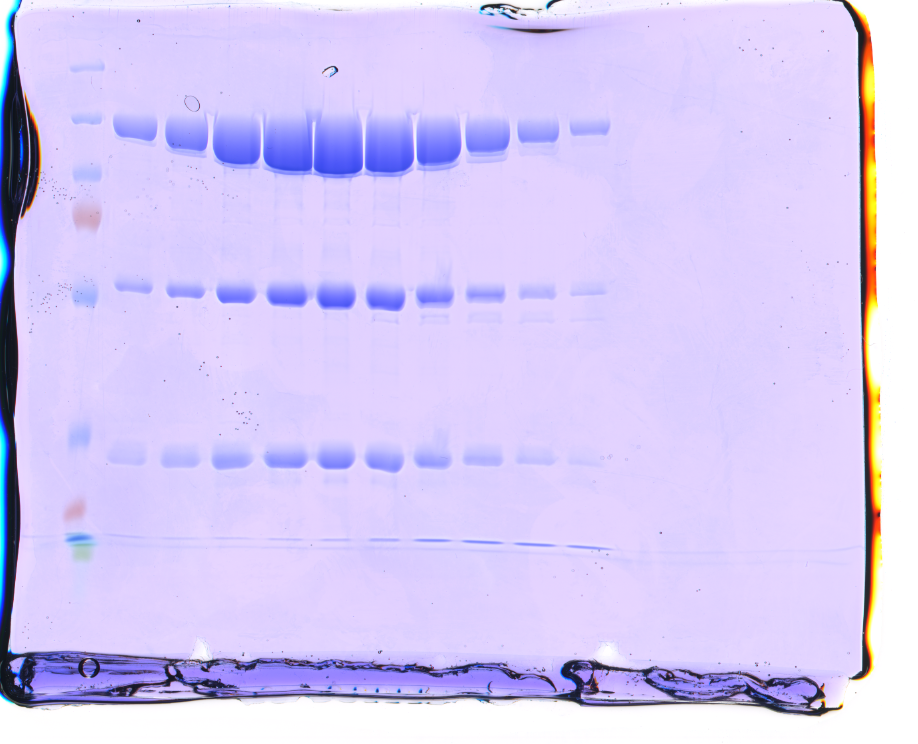

Supplement: Supplementary file 1 [file LSA-2024-02899_SdataF2_F3_F4_F5.zip › original data/Fig 5B _ Jet purification full gel.tif]
